# Animal Rabies Surveillance, China, 2004–2018

**DOI:** 10.3201/eid2612.200303

**Published:** 2020-12

**Authors:** Ye Feng, Yuyang Wang, Weidi Xu, Zhongzhong Tu, Tingfang Liu, Minghe Huo, Yan Liu, Wenjie Gong, Zheng Zeng, Wen Wang, Yinhong Wei, Changchun Tu

**Affiliations:** Academy of Military Medical Sciences, Changchun, China (Y. Feng, Y. Wang, W. Xu, Z. Tu, T. Liu, M. Huo, Y. Liu, W. Gong, C. Tu);; Jilin Agricultural University, Changchun (W. Xu, T. Liu);; Jilin University College of Veterinary Medicine, Changchun (M. Huo, W. Gong);; Center for Animal Disease Control and Prevention of Chongqing, Chongqing, China (Z. Zeng);; Animal Health Inspection Institute of Xinjiang Uygur Autonomous Region, Urumqi, China (W. Wang);; Center for Animal Disease Control and Prevention of Alxa Youqi, Alxa, China (Y. Wei);; Jiangsu Co-Innovation Center for the Prevention and Control of Important Animal Infectious Disease and Zoonoses, Yangzhou University, Yangzhou, China (C. Tu)

**Keywords:** rabies virus, surveillance, genetic diversity, transboundary transmission, viruses, China

## Abstract

Rabies is a severe zoonotic disease in China, but the circulation and distribution of rabies virus (RABV) within animal reservoirs is not well understood. We report the results of 15 years of surveillance of the first Chinese Rabies Surveillance Plan in animal populations, in which animal brain tissues collected during 2004–2018 were tested for RABV and phylogenetic and spatial–temporal evolutionary analyses performed using obtained RABV sequences. The results have provided the most comprehensive dataset to date on the infected animal species, geographic distribution, transmission sources, and genetic diversity of RABVs in China. In particular, the transboundary transmission of emerging RABV subclades between China and neighboring countries was confirmed. The study highlights the importance of continuous animal rabies surveillance in monitoring the transmission dynamics, and provides updated information for improving current control and prevention strategies at the source.

Rabies is a fatal zoonotic disease of humans and almost all warm-blooded animals, causing severe dysfunction of the central nervous system (*1*). About 99% of human cases occur in developing countries, mainly in Asia and Africa (*2*). Rabies is a major public issue throughout China, resulting in several hundred human deaths every year during 2015–2018 (*3*). More than 95% of human rabies cases are caused by rabid dogs (*4*). In contrast, the numbers of animal rabies cases reported in China are much lower than those of humans; only several provinces, autonomous regions, or municipalities report animal rabies cases to national veterinary authorities, as disseminated by the Veterinary Bulletin, the only official journal to report monthly information on animal infectious diseases in China (*5*). Even so, such scattered studies have still shown an increase in wildlife rabies in red foxes (*Vulpes vulpes*), raccoon dogs (*Nyctereutes procyonoides*), and ferret badgers (*Melogale moschata* in the mainland and *Melogale moschata subaurantiaca* in Taiwan). Rabies in dogs and livestock has also increased and expanded geographically to include Heilongjiang, Inner Mongolia, Xinjiang, Qinghai, Tibet, and Taiwan, provincial regions within which rabies had rarely or never been reported previously (*6**–**11*). These investigations had monitored the emergence of fox- and raccoon dog–specific RABVs in north China that caused the outbreaks in livestock; some wildlife isolates shared a high nucleotide identity with those circulating in neighboring countries (*6*,*7*,*10*). This similarity is a matter for concern because China is surrounded by 14 contiguous countries, all of which are rabies endemic and within which the genetic diversity and phylogenetic characteristics of RABVs have not been well studied.

An understanding of the status of animal rabies is a prerequisite for control and possible elimination of human rabies. Since 2004, China has implemented annual surveillance of animal rabies, with the National Reference Laboratory for Animal Rabies at the Institute of Military Veterinary Medicine, (Changchun, Jilin Province, China) as the project leader (*12*). This surveillance focuses mainly on dogs, cats, livestock, and wild animal reservoirs that have the potential to maintain the circulation and transmission of RABVs in China. As part of this program, using the epidemiologic information collected and nucleoprotein (N) gene sequences of RABV isolates obtained during 2004–2018, we investigated the infected animal species, geographic distribution, animal sources, and genetic diversity of RABVs in China, as well as their phylogenetic and phylogeographic relationships with those of neighboring countries. Our objective was to provide updated information about the animal rabies situation and its public health impact in China and neighboring countries.

## Methods

### Sample Collection and Detection of Rabies Virus

Since 2004, the Ministry of Agriculture and Rural Affairs of China has implemented the Rabies Surveillance Plan with a focus on free-roaming and stray dogs and cats, especially those showing abnormal behaviors such as biting humans. The plan also requires the monitoring of suspected rabies outbreaks in livestock and wild animals. During 2004–2018, brain tissues of 185 animals suspected of having rabies (dead dogs, dogs behaving strangely or biting humans, livestock showing rabieslike clinical signs, dead foxes, wolves, and raccoon dogs) were submitted (Appendix Table 1). In addition, 10,118 brain tissues were collected for active surveillance from 3 types of apparently healthy dogs, mostly from rabies-endemic rural areas: free-roaming and ownerless dogs, slaughtered dogs (for meat consumption), and dogs killed during emergencies (culled in rabies outbreak areas to prevent further transmission) (Appendix Table 2). All specimens were collected and submitted to the reference laboratory by the regional Centers of Animal Disease Prevention and Control.

The brain tissues were examined by the direct fluorescent antibody test (FAT) using FITC-conjugated anti-rabies monoclonal antibody (Fujirebio Diagnostics Inc., https://www.fujirebio.com) (*13*). To obtain the complete coding sequence of the N gene, rabies-positive specimens were subjected to RNA extraction using TRIzol Reagent (Invitrogen, https://www.thermofisher.com), followed by reverse transcription PCR with the SuperScript III First-Strand Synthesis System and the Platinum Taq DNA Polymerase High Fidelity kit (Invitrogen) (*6*).

### Gene Sequencing and Phylogenetic Analysis

Amplified N gene sequencing was performed commercially by the Sanger method and submitted to GenBank (see Appendix Table 3 for accession numbers). Phylogenetic analysis of the complete N gene was performed on these sequences and on representative sequences retrieved from GenBank, covering samples collected in China and its neighboring countries from the 1940s through 2018 (Appendix Table 3). The MEGA 7 program package was used to construct the phylogenetic trees using the neighbor-joining method with 1,000 bootstrap replicates (*14*). Trees were visualized using Figtree version 1.4.2 (http://tree.bio.ed.ac.uk/software/figtree).

To rank the prevalence of the different RABV phylogroups and to analyze their transmission trends in China, we retrieved the sequences of all RABV strains from China deposited in GenBank. After we removed duplicate sequences and those without clear time information, we phylogenetically classified the remaining sequences, along with those obtained during this study, by the procedure described previously and chronologically sorted them by collection date.

### Spatial–Temporal Evolutionary Analysis

To investigate the temporal signal and clock likeness of molecular phylogenies based on the N gene dataset, the linear evolutionary rates of different RABV clades were estimated using the Bayesian Markov chain Monte Carlo in BEAST version 1.8.2 package (*15*,*16*). For these analyses, we selected the general time reversible model as the substitution model and gamma plus invariable sites as the site heterogeneity model based on the calculations of Model Generator (*17*,*18*). An uncorrelated log normal relaxed molecular clock model and the constant size model as a coalescent tree prior were also selected for the analyses, which were run for 100 million steps with sampling at every 10,000 states (*19*). The BEAGLE parallel computation library was used to enhance the speed of the likelihood calculations (*20*). Finally, the resulting log file was checked using TRACER version 1.5 (http://tree.bio.ed.ac.uk/software/tracer) to confirm that all effective sample sizes were >200. The tree file was obtained using TreeAnnotator version 1.8.2 with a burn-in of 10%, and the maximum clade credibility tree was visualized by FigTree version 1.4.2 (*16*). Based on the analyses, estimations were made of the rates of nucleotide substitution and the time to most recent common ancestor (tMRCA) for each RABV clade.

To investigate the phylogeographic spread of RABVs in China and neighboring countries, we used a Bayesian stochastic search variable selection (BSSVS) approach to analyze transmission routes of RABV subclades, in which we applied a Bayes factor to determine the best supported transmission event between 2 geographic locations. Bayes factors were calculated by SpreaD3 software with a value >3 as cutoff (*21*,*22*).

## Results

### Current Animal Rabies Situation in China

During 2004–2018, animal brain tissues collected from 185 animals with suspected rabies in 17 provinces were submitted to our laboratory; 144 of them (77.8%) were confirmed by FAT as rabies virus positive (Appendix Table 1). Among the positive species, dogs were the main infected animals, accounting for 68.8% of total cases (99/144), followed by cattle (12.5%), sheep (9.7%), camels (4.2%), foxes (2.1%), pigs (1.4%), raccoon dogs (0.7%), and donkeys (0.7%) (Appendix Table 1). Concurrently, 33 (0.33%) of 10,118 brain tissue samples taken during an active surveillance of apparently healthy dogs from 7 provinces across China were found to be FAT-positive. Of the 33 positive specimens, 31 were from free-roaming and ownerless dogs (including stray dogs) and 2 were from dogs killed during an emergency (Appendix Table 2). For livestock rabies, 29 cattle, sheep, and camel cases were reported in Inner Mongolia and Xinjiang during 2013–2018, all of which were caused by fox bites (Appendix Table 1).

### Phylogenetic Analysis and Evolution of Animal RABVs in China

**Figure 1:**
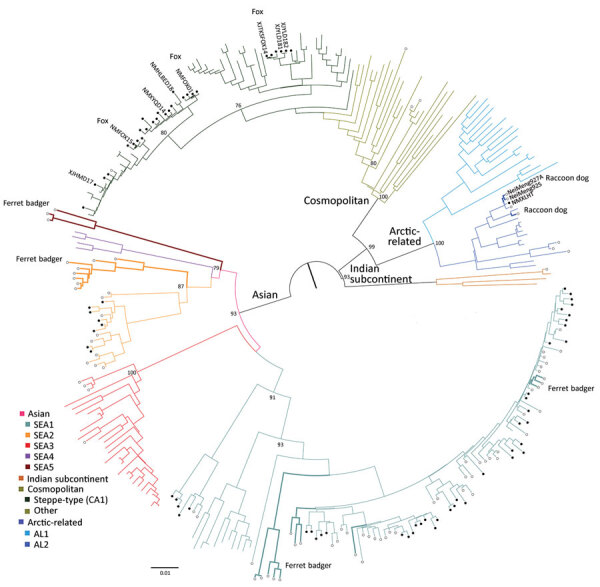
Phylogenetic analysis of 300 full rabies virus nucleoprotein sequences showed that RABVs in China could be classified into 4 major clades and 8 subclades. Bootstrap values = 1,000. Solid circles indicate sequences from this study; open circles indicate representative sequences from China previously published and retrieved from GenBank (Appendix Table 3). Unlabeled sequences are from surrounding countries; a few are vaccine sequences in the subclade of another Cosmopolitan clade. Named branches: dog isolates spilling out from wild animals; bold branches: wild animal isolates as indicated. SEA, Southeast Asia.

**Figure 2:**
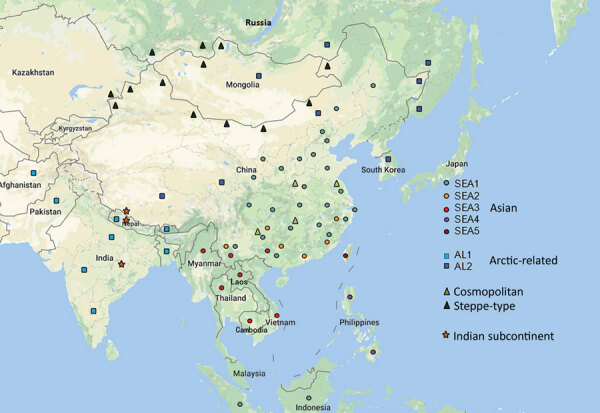
Geographic distribution of rabies virus clades and subclades in China and neighboring countries. The sequence information is from this study and GenBank (Appendix Table 3). SEA, Southeast Asia.

A total of 108 complete N genes were amplified from 177 positive brain tissue samples. Of these, we selected 78, representing different years, animal species, outbreaks, and locations, together with 222 reference sequences from China, as well as from neighboring and other countries, to determine their phylogenetic characteristics (Appendix Table 3). Animal RABVs in China were clustered within 4 major clades: Asian, Cosmopolitan, Arctic-related, and Indian Subcontinent, together with different subclades (Figure 1). The Asian clade, the most prevalent one, widely distributed throughout China and Southeast Asia (SEA) countries, shows abundant genetic diversity and is transmitted mainly by dogs. This lineage was further divided into 5 subclades and different lineages. SEA1, 2, 3, and 5 subclades circulate mainly in populous areas within China; however, some lineages and strains in SEA1 and 3 were also found circulating in some countries in Southeast Asia, whereas SEA4 was restricted to the Philippines (Figure 2). Reported only in China, ferret badger RABVs were found to have abundant genetic diversity and were segregated into different lineages within SEA1, 2, and 5 (Figure 1) (*23*). Within the Cosmopolitan clade, which includes some vaccine strains, a steppe-type subclade was fox-transmitted and distributed along north and northwest border areas and neighboring Mongolia, Russia, and Kazakhstan, forming 2 major lineages (Figure 1). Other Cosmopolitan strains were dog-transmitted and mainly distributed in populous inland areas (Figure 2). Arctic-related RABVs in China segregated within the AL2 subclade and formed 2 lineages, one in northeastern China and far eastern Russia, Mongolia, and South Korea with dogs and raccoon dogs as the major hosts, and the other in southwestern China with dogs as the major transmission source (Figures 1,2). The Indian Subcontinent clade had not been identified in China until the first human rabies case caused by this clade was identified in 2017 in the border area of Tibet close to Nepal (*24*). That human case was caused by the bite of a local stray dog and remains the only Indian Subcontinent clade RABV confirmed so far in China.

**Figure 3:**
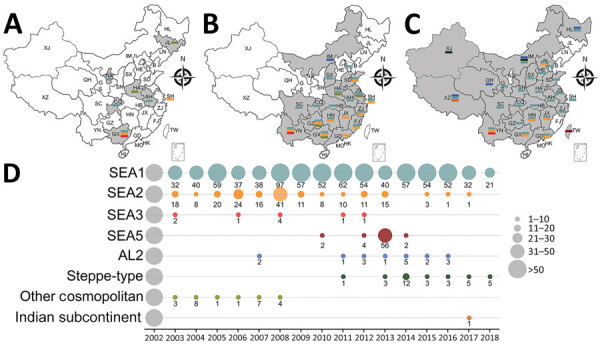
Spatial–temporal dynamics of RABVs in China. Phylogenetic analysis of 1,118 sequences representing 1,118 rabies cases or virus strains, including those obtained in this study using different gene fragments, followed by chronological summation of each subclade. A–C) Distribution of identified subclades during 3 time periods: A) before 2004; B) 2004–2008; C) 2009–2018. D) Quantitative trends of 8 Chinese RABV subclades during 2004–2018. Exact numbers within each subclade are given below the circles. SEA, Southeast Asia.

For the chronological sorting of different subclades, we retrieved all 2,486 RABV sequences from China deposited in GenBank. After removing repeated sequences and those without date information, 1,118 eligible sequences remained, representing 1,118 Chinese strains isolated during 1969–2018 (including those in Figure 1). These sequences included complete genome (n = 45), full length or partial N (n = 819), glycoprotein (G; n = 208), phosphoprotein (P; n = 15), matrix protein (M; n = 25) and RNA-dependent RNA polymerase (L; n = 6) genes. Figure 3 shows the spatial–temporal trends of different RABV subclades in China, in which the 55 Chinese RABV sequences submitted to GenBank between 1969 (the earliest submission) and 2003 (therefore listed chronologically as “before 2004”) segregated within 3 SEA and 1 Cosmopolitan subclade. Following initiation of official rabies surveillance in 2004, numbers of sequences submitted to GenBank sharply increased and high numbers of submissions have been maintained thereafter. The resulting data showed clearly that most rabies outbreaks have been caused by strains of the Asian clade (93.3%), with limited involvement of strains of the other 3 clades. Within the Asian clade, the subclade SEA1 predominated in rabies endemics in China (70.1%), followed by SEA2 (16.7%). SEA1 is the most widely distributed of the subclades and continues to spread. The steppe-type subclade first emerged in 2011 and has rapidly become predominant among the livestock RABV strains found along border areas in Inner Mongolia and Xinjiang Province (Figure 2). AL2 was first detected in 2007 and has become a common subclade in recent years (*10*). The Indian Subcontinent clade caused an occasional case in 2017. The result showed that steppe-type, AL2, and Indian Subcontinent strains are emerging RABVs in China. Dog-transmitted Cosmopolitan strains have not been detected during the past decade.

### Transmission of Animal Rabies in China and Neighboring Countries

**Figure 4:**
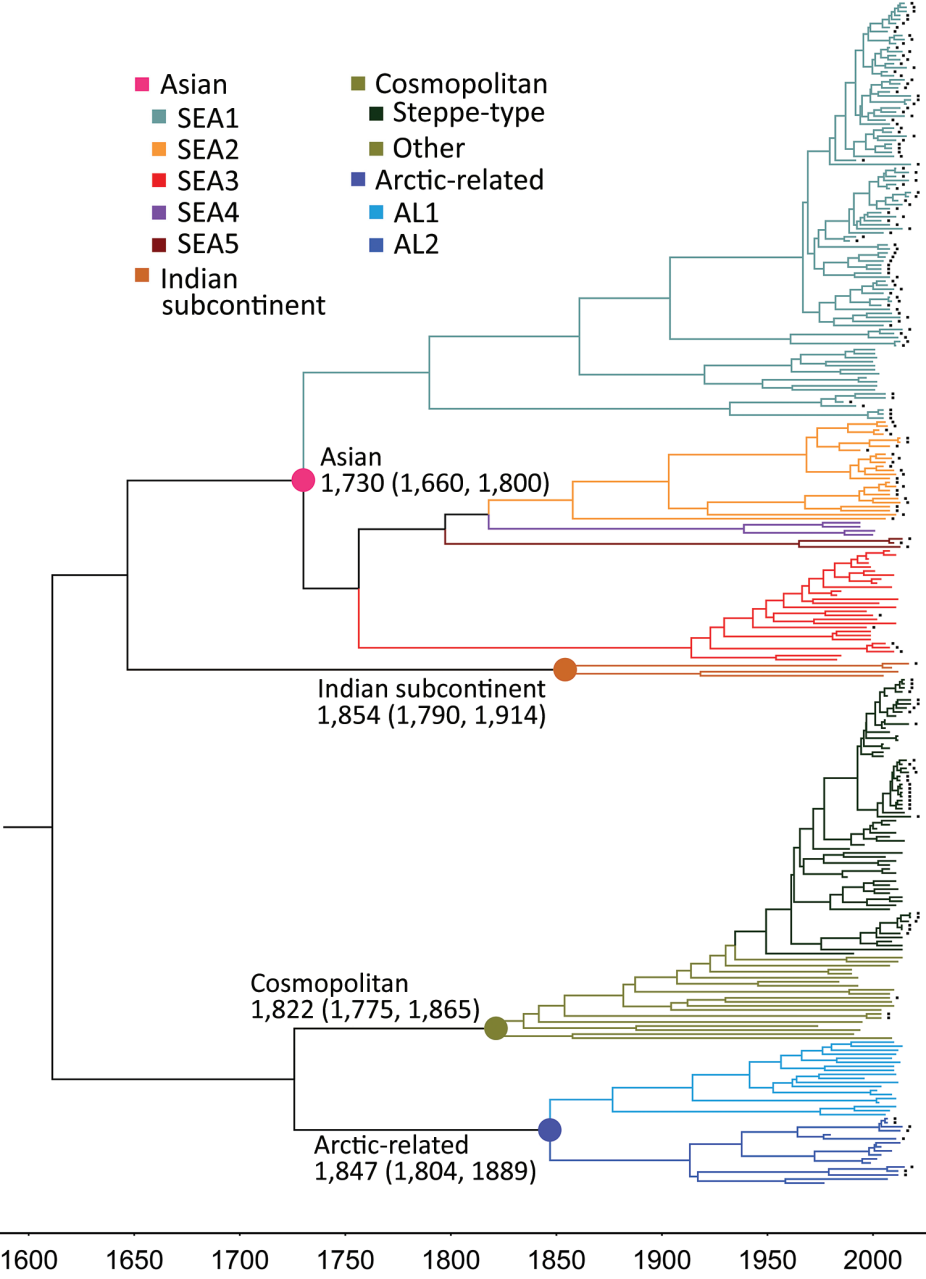
Nucleoprotein gene–based maximum clade credibility tree of rabies viruses. The estimated time to most recent common ancestor of these clades and their 95% highest posterior density values are indicated. The same sequences as in Figure 1 were used, except for those of 5 vaccine strains listed at end of Appendix Table 3. Black solid squares indicate strains from China. SEA, Southeast Asia.

**Figure 5:**
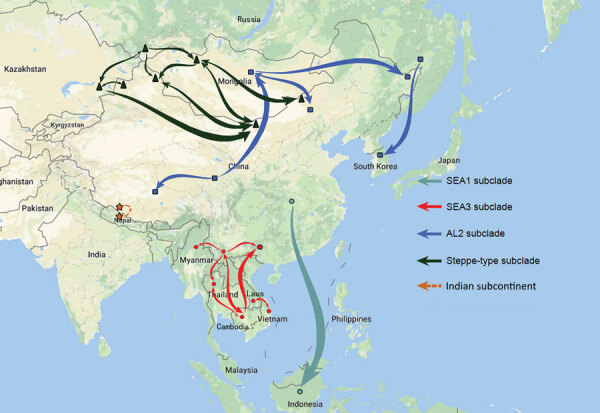
Proposed transboundary transmission of rabies viruses between China and neighboring countries determined by the Bayesian stochastic search variable selection approach. Unbroken lines: transmission events with a Bayes factor >3; broken line: transmission event with a Bayes factor <3. SEA, Southeast Asia.

Results of the Bayesian skyline model analysis showed that the mean rate of nucleotide substitution for the tested RABVs was 3.50 × 10^−4^ substitutions per site per year (95% highest posterior density 2.90–4.11 × 10^−4^ substitutions per site per year). This finding is consistent with the previous analyses of evolutionary change performed on dog-related RABV N genes (*25*). Differences in evolutionary rates among the clades and subclades were not significant. All representative RABVs in China and neighboring countries shared a tMRCA, predicted to merge 349–563 years ago (Figure 4). Further analysis of transmission routes of RABV subclades by BEAST revealed the transboundary transmissions of rabies in different border regions around China. The significant translocation pathway of SEA1 (Bayes factor 76.9) (Appendix Table 4) from China to Indonesia was identified in accordance with our previous analysis of the G gene (Figure 5) (*26*). Moreover, many SEA3 strains in Myanmar, Thailand, Laos, and Vietnam were genetically close to some strains circulating in Yunnan and Guangxi, China, indicating mutual transmission of SEA3 strains between China and bordering SEA countries (Bayes factor 4.3–85.0), as discussed previously (*27*). The same transmission was also found for steppe-type and AL2 subclades in border regions between China and Kazakhstan, Russia, Mongolia, and South Korea (Bayes factor 3.17–229.87). The most noteworthy event was the recent cross-border transmission of an Indian subcontinent strain from Nepal to the border region of Tibet, albeit with a lower Bayes factor (0.9), which caused a human rabies death in 2017 (*24*).

## Discussion

There have been studies of the genetic diversity and transmission dynamics of RABVs in China, but the background information was compiled mainly from RABVs collected before 2010 or restricted to several provinces or geographic regions (*26*,*28**–**30*). Information about the molecular epidemiology of RABV within the past 10 years has been lacking, particularly within the context of the recently increasing animal rabies situation in the north, northwest, northeast, and southwest regions of China (*6*,*7*,*31*,*32*). In addition, although all the neighboring countries of China are rabies endemic, phylogenetic relationships and transboundary transmission of RABVs between China and these countries have not been systematically investigated; however, a 2013 study based on N gene sequences of RABV isolates collected before 2010 concluded that national borders effectively halted transboundary rabies transmission from China (*33*). Our study, however, has provided the most comprehensive update of RABV genetic diversity and transmission dynamics in China and has systematically compared these characteristics with those of neighboring countries, using many recent sequences obtained in our continuous surveillance during 2004–2018, along with many representative sequences from GenBank published in the past decade. The results have not only revealed the abundant genetic diversity of RABVs from China with many lineages or strains in most subclades genetically close to those circulating in neighboring countries (Figure 1) but also delineated the phylogeographic distribution of diverse RABVs in China and neighboring countries (Figure 2). The results have revealed 2 epidemic modes existing in China. The first is the historical dog-mediated rabies epidemic in populous inland provinces mainly in the center, east, and south, in which subclades within the Asian clade, particularly SEA1 followed by SEA 2, play dominant roles. The second consists mainly of outbreaks caused by the emerging subclades AL2, steppe-type, and Indian Subcontinent that have been closely associated over the past decade with cross-border transmission (Figure 5). As determined by analysis of data with a Bayes factor >3 using the BSSVS approach, fox-transmitted steppe-type viruses circulate in north and northwest border areas in Inner Mongolia and Xinjiang Province, with transboundary transmission between China and Mongolia, Russia, and Kazakhstan. Wild foxes have become the main rabies transmitter in these areas (Figure 1; Appendix Table 1). The raccoon dog–transmitted AL2 subclade emerged in the northeast likely through cross-border transmission from Mongolia. The Indian Subcontinent subclade, emerging to cause a human death in Tibet in 2017, is the most recent transboundary transmission event of dog-mediated rabies from a neighboring country (*24*). Our study has also shown transboundary transmission of the SEA3 subclade, mediated by dogs in the border areas between southwest China and SEA countries (Figure 5). 

Wild animals remain the major sources of AL2 and steppe-type subclades and usually transmit the viruses causing human and livestock rabies in the steppes of Mongolia (*34*,*35*). Surprisingly, however, the surveillance in our study identified the initial spillover of these 2 subclades into dogs within China. An AL2 strain (NMXLHT) was isolated from an infected dog in 2013 in Inner Mongolia (Appendix Table 1) and grouped together with the first 2 AL2 strains (NeiMeng 927 and 925) isolated from rabid raccoon dogs in 2007 in Inner Mongolia (Figure 1) (*10*). Their collection sites were ≈200 km apart. Two steppe-type isolates (NMXYQD14 and XJHMD17) were also identified from dogs: the first in Inner Mongolia in 2014 and the second in 2017 in Xinjiang (Appendix Table 1). These dogs had exhibited strange behavior and had bitten some humans or other dogs. In 2018, another 3 dog steppe-type isolates (NMHLBED18, XJYLD181, and XJYLD182) were detected, 1 from a dog suspected of having rabies in Inner Mongolia (Appendix Table 1) and 2 from apparently healthy dogs in Xinjiang Province (Appendix Table 2). All 5 of these dog isolates had a very close phylogenetic relationship with 3 fox isolates (NMFOX01, NMFOX15, and XJTKSFOX14) (Figure 1). A case of fox-mediated human rabies was diagnosed by reverse transcription PCR in Xinjiang Province in 2016, although the causative virus was not sequenced (*9*). These results not only demonstrated the spillover of wildlife RABVs into dogs in the past decade but also indicated that the risk of the spillover is increasing and threatening public health in northern China.

Rabies is still neglected in China, and efforts to increase awareness and strengthen control measures at the animal sources are still insufficient. As a consequence, the number of animal rabies cases officially reported during 2004–2018 (no data are available from before 2004) was only 893 (*5*), a much lower figure than the 25,424 human cases reported in China over the same period (*3*). Of the reported animal rabies cases, only a small proportion was submitted for laboratory diagnosis, and the 185 rabies-suspected animals tested in our study account for most of these. This low figure notwithstanding, 15 years of continuous surveillance have been adequate to reveal the spread of animal rabies (Figure 3) and have highlighted that dog rabies is still widely distributed, accounting for 74.6% (132/177) of total infected animals (Appendix Tables 1, 2). Phylogenetic analyses (Figure 1) have clearly shown that all livestock RABV isolates grouped together with either dog or fox isolates, indicating that dogs and foxes are major transmission sources. These analyses have also shown that some RABV isolates were ferret badger specific, circulating solely in ferret badgers and forming independent lineage (within SEA 2) or even a sublcade (such as SEA 5 in Taiwan). Moreover, the ongoing surveillance has also revealed the spillover of fox- and raccoon dog–transmitted RABVs into dogs, which emphasizes the importance of sequence-based analysis in tracking the sources of animal rabies cases, for which investigation into the retrospective biting history is impossible. In addition, our study has delineated the current status of wildlife rabies in China, emphasizing the roles of the relevant wild reservoir hosts in the current increase of rabies transmission. Altogether, our work has shown that sustained surveillance of animal rabies, combined with sequence-based analysis of collected RABVs, is a robust strategy to track the transmission source.

In conclusion, although animal rabies is largely underreported in China, our continuous surveillance has been able to document the current status and transmission trends of animal rabies within the country, showing that these consist of a combination of historical dog-mediated rabies in populous inland areas and the emergence of wildlife-mediated rabies during the past decade in border areas. We have also completely updated the phylogenetic and phylogeographic characteristics of RABVs in China, with particular attention to the prevalence and transboundary transmission of emerging RABV subclades.

Appendix Additional information on animal rabies surveillance in China.
