# Supplementary material for: Animal Rabies Surveillance, China, 2004–2018
Source: Emerg Infect Dis. 2020 Dec;26(12):2825–34. doi: 10.3201/eid2612.200303 (PMC7706947; doi:10.3201/eid2612.200303)
Supplement: Appendix — Additional information on animal rabies surveillance in China. [file 20-0303-Techapp-s1.pdf]

Article DOI: https://doi.org/10.3201/eid2612.200303

Animal Rabies Surveillance, China, 2004–2018

Appendix

Appendix Table 1. Detection of suspected rabies in brain samples submitted during 2004–2018 (positive/total)*

Province	Dog	Cattle	Sheep	Camel	Pig	Donkey	Wolf†	Fox	Raccoon dog	Total
CQ	57/70	–	–	–	–	–	0/2	–	–	57/72
HN	6/7	–	–	–	2/2	–	–	–	–	8/9
SD	–	2/2	–	–	–	–	–	–	–	2/2
TJ	4/5	2/2	–	–	–	–	–	–	–	6/7
GD	2/5	–	–	–	–	–	–	–	–	2/5
SX	10/11	1/1	3/3	–	–	1/1	–	–	–	15/16
HE	0/1	–	–	–	–	–	–	–	–	0/1
JS	2/2	–	–	–	–	–	–	–	–	2/2
YN	0/1	–	–	–	–	–	–	–	–	0/1
SH	9/12	–	–	–	–	–	–	–	–	9/12
IM‡	3/3	11/14	5/5	4/4	–	–	–	2/3	1/1	26/30
GS	1/3	–	–	–	–	–	–	–	–	1/3
XJ‡	1/1	1/2	6/8	2/2	–	–	–	1/1	–	11/14
SN	0/2	–	–	–	–	–	–	–	–	0/2
HLJ	0/1	1/1	–	–	–	–	–	–	–	1/2
ZJ	4/6	–	–	–	–	–	–	–	–	4/6
LN	0/1	–	–	–	–	–	–	–	–	0/1
Total	99/131	18/22	14/16	6/6	2/2	1/1	0/2	3/4	1/1	144/185 (77.84%)

*CQ, Chongqing; GD, Guangdong; GS, Gansu; HA, Henan; HE, Hebei; HLJ, Heilongjiang; HN, Hunan; IM, Inner Mongolia; JS, Jiangsu; LN, Liaoning; SD, Shandong; SH, Shanghai; SN, Shaanxi; SX, Shanxi; TJ, Tianjin; XJ, Xinjiang; YN, Yunnan; ZJ, Zhejiang.

†Two dead wolves were submitted by a zoo.

‡In IM and XJ, 3 dog rabies cases (2 in IM and 1 in XJ) and all livestock and fox rabies cases were caused by a steppe-type subclade during 2013–2018, with foxes being transmission sources. In IM, the remaining dog rabies case and raccoon dog rabies case were caused by AL2 subclade (Figure 1).

Appendix Table 2. Detection of healthy-looking dogs for surveillance during 2004–2018 (positive/total)*

Province	Free-roaming and ownerless dogs	Emergently killed dogs	Slaughtered dogs	Total
GD	7/6085	–	0/265	7/6350
HN	6/1211	2/41	–	8/1252
SN	–	0/112	–	0/112
GX	16/1175	–	0/145	16/1320
ZJ	0/6	–	–	0/6
XJ	2/988†	–	0/7	2/995
QH	0/83	–	–	0/83
Total	0.32% (31/9,548)	1.3% (2/153)	0/417	0.33% (33/10118)

*GD, Guangdong; GX, Guangxi; HN, Hunan; QH, Qinghai; SN, Shaanxi; XJ, Xinjiang; ZJ, Zhejiang.

†These 2 dog rabies cases were caused by steppe-type subclade, according to the phylogenetic analysis in Figure 1.

Appendix Table 3. Information on 300 full N gene sequences of RABVs used in the phylogenetic analyses

Country	Location	Virus name	Species	Year	Phylogenetic clade/subclade	GenBank accession no.
Afghanistan		04027AFG	Dog	1996	Arctic-related/AL1	EU086162
Afghanistan		02052AFG	Dog	2002	Arctic-related/AL1	KX148225
Afghanistan		04029AFG	Dog	2004	Arctic-related/AL1	KX148227
Afghanistan		09032AFG	Dog	2009	Arctic-related/AL1	JX987739
Afghanistan		BDR6	Cattle	2010	Arctic-related/AL1	AB699218
Afghanistan		ACS11	Human	2011	Arctic-related/AL1	KF992019
Bhutan		Btn115	Cattle	2010	Arctic-related/AL1	AB910532
Brazil		95022BRE	Dog	1995	Cosmopolitan/other	KX148215
Cambodia		9914CBG	Dog	1997	Asian/SEA3	EU086170
Cambodia		9911CBG	Dog	1998	Asian/SEA3	EU086168
Cambodia		I0515204	Dog	1998	Asian/SEA3	KM366251
Cambodia		9908CBG	Dog	1999	Asian/SEA3	EU086167
Cambodia		M0516129	Dog	2002	Asian/SEA3	KM366209
Cambodia		N1011101	Dog	2003	Asian/SEA3	KM366270
Cambodia		O0405536	Dog	2004	Asian/SEA3	KM366206
Cambodia		S1030654	Dog	2008	Asian/SEA3	KM366202
Cambodia		T1002618	Dog	2009	Asian/SEA3	KM366262
Cambodia		U0821622	Dog	2010	Asian/SEA3	KM366267
Cambodia		V0808656	Dog	2011	Asian/SEA3	KM366221
China*	Chongqing	CQWSD01	Dog	2005	Asian/SEA1	KT221095
China*	Chongqing	CQWXD01	Dog	2005	Asian/SEA1	KT221093
China*	Chongqing	CQQJD6	Dog	2006	Asian/SEA1	KT221097

Country	Location	Virus name	Species	Year	Phylogenetic clade/subclade	GenBank accession no.
China*	Chongqing	CQWLD02	Dog	2006	Asian/SEA1	KT221094
China*	Chongqing	CQFJD02	Dog	2007	Asian/SEA1	KT894569
China*	Chongqing	CQBSD01	Dog	2008	Asian/SEA1	KT894564
China*	Chongqing	CQYBD01	Dog	2008	Asian/SEA1	KT221098
China*	Chongqing	CQWLD03	Dog	2009	Asian/SEA1	KT894559
China*	Chongqing	CQD023	Dog	2010	Asian/SEA1	KT894560
China*	Chongqing	CQCSD01	Dog	2011	Asian/SEA1	KT894563
China*	Chongqing	CQJLPD01	Dog	2011	Asian/SEA1	KT894567
China*	Chongqing	CQQJD11	Dog	2011	Asian/SEA1	KT894562
China*	Chongqing	CQWSD11	Dog	2011	Asian/SEA1	KT894561
China*	Chongqing	ZDW046	Dog	2011	Asian/SEA1	KT894571
China*	Chongqing	CQJLPD13	Dog	2013	Asian/SEA1	KT894568
China*	Chongqing	CQCSD17	Dog	2017	Asian/SEA1	MG383887
China*	Chongqing	CQJJD17	Dog	2017	Asian/SEA1	MG383888
China*	Chongqing	CQYCD17	Dog	2017	Asian/SEA1	MK124736
China*	Chongqing	CQYCD18	Dog	2018	Asian/SEA1	MN784133
China*	Gansu	GSHSD14	Dog	2014	Asian/SEA1	KT221103
China*	Guangxi	GXBSD01	Dog	2009	Asian/SEA1	KT894576
China*	Guangxi	GXQZD01	Dog	2009	Asian/SEA2	KT221107
China*	Guangxi	GXRXD01	Dog	2010	Asian/SEA1	KT894575
China*	Guangxi	GXWXD01	Dog	2013	Asian/SEA2	KT894577
China*	Guangxi	GXWXD02	Dog	2013	Asian/SEA2	KT894578
China*	Heilongjiang	HLJC16	Cattle	2016	Asian/SEA1	MG383884
China*	Hunan	HN05	Dog	2005	Asian/SEA2	KT894558
China*	Hunan	HuND02	Dog	2005	Asian/SEA1	KT221100
China*	Hunan	HuND28	Dog	2005	Asian/SEA1	KT221101
China*	Hunan	HN06	Dog	2006	Asian/SEA1	KT894557
China*	Hunan	HNP02	Pig	2006	Asian/SEA2	KT221102
China*	Hunan	HuNPN01	Pig	2006	Asian/SEA2	DQ496219
China*	Hunan	HNYZD01	Dog	2010	Asian/SEA2	KT894572
China*	Hunan	HNNYD03	Dog	2011	Asian/SEA2	KT894573
China*	Inner Mongolia	NMC01	Cattle	2013	Cosmopolitan/ST	KJ152772
China*	Inner Mongolia	NMSH01	Sheep	2013	Cosmopolitan/ST	KJ152774
China*	Inner Mongolia	NMAYC01	Cattle	2014	Cosmopolitan/ST	KJ737435
China*	Inner Mongolia	NMAYCAM03	Camel	2014	Cosmopolitan/ST	KX533959
China*	Inner Mongolia	NMC02	Cattle	2014	Cosmopolitan/ST	KJ748634
China*	Inner Mongolia	NMC03	Cattle	2014	Cosmopolitan/ST	KJ748635

Country	Location	Virus name	Species	Year	Phylogenetic clade/subclade	GenBank accession no.
China*	Inner Mongolia	NMC04	Cattle	2014	Cosmopolitan/ST	KJ748636
China*	Inner Mongolia	NMCAM01	Camel	2014	Cosmopolitan/ST	KJ748631
China*	Inner Mongolia	NMCAM02	Camel	2014	Cosmopolitan/ST	KJ748632
China*	Inner Mongolia	NMFOX01	Fox	2014	Cosmopolitan/ST	KJ748633
China*	Inner Mongolia	NMXLHT	Dog	2014	Arctic-related/AL2	KU041697
China*	Inner Mongolia	NMXYQD14	Dog	2014	Cosmopolitan/ST	KT894582
China*	Inner Mongolia	NMCAM15	Camel	2015	Cosmopolitan/ST	KX533961
China*	Inner Mongolia	NMFOX15	Fox	2015	Cosmopolitan/ST	KX533960
China*	Inner Mongolia	NMHLBEC16	Cattle	2016	Cosmopolitan/ST	KX533963
China*	Inner Mongolia	NMXYQC16	Cattle	2016	Cosmopolitan/ST	KX533962
China*	Inner Mongolia	NMHLBES17	Sheep	2017	Cosmopolitan/ST	MG383886
China*	Inner Mongolia	NMALSS18	Sheep	2018	Cosmopolitan/ST	MK124741
China*	Inner Mongolia	NMHLBED18	Dog	2018	Cosmopolitan/ST	MK124739
China*	Jiangsu	JSWXD18	Dog	2018	Asian/SEA1	MK124742
China*	Shandong	SDJNC02	Cattle	2007	Asian/SEA1	KT894585
China*	Shanghai	SHCMD01	Dog	2010	Asian/SEA1	KT894579
China*	Shanghai	SHPDD01	Dog	2011	Asian/SEA1	KT894581
China*	Shanghai	SHPDD02	Dog	2013	Asian/SEA1	KT894580
China*	Shanghai	SHBSD16	Dog	2016	Asian/SEA1	MG383885
China*	Shanghai	SHJDD18	Dog	2018	Asian/SEA1	MN784135
China*	Shanxi	SXLFD03	Dog	2007	Asian/SEA1	KT221111
China*	Shanxi	SXD01	Dog	2008	Asian/SEA1	KT221109
China*	Shanxi	SXDTD01	Dog	2010	Asian/SEA1	KT221108
China*	Shanxi	SXDTD13	Dog	2013	Asian/SEA1	KT894584
China*	Shanxi	SXYL15	Cattle	2015	Asian/SEA1	KR230090
China*	Tianjin	TJD03	Dog	2008	Asian/SEA1	KT221106
China*	Tianjin	TJC14	Cattle	2014	Asian/SEA1	KT894574
China*	Xinjiang	XJTCS01	Sheep	2013	Cosmopolitan/ST	KJ152773
China*	Xinjiang	XJTKSFOX14	Fox	2014	Cosmopolitan/ST	KU041696
China*	Xinjiang	XJTKSS02	Sheep	2014	Cosmopolitan/ST	MK321229
China*	Xinjiang	XJFYCA15	Camel	2015	Cosmopolitan/ST	MG383882
China*	Xinjiang	XJHMS16	Sheep	2016	Cosmopolitan/ST	MK321230
China*	Xinjiang	XJHMD17	Dog	2017	Cosmopolitan/ST	MK124737
China*	Xinjiang	XJYLC17	Cattle	2017	Cosmopolitan/ST	MK124738
China*	Xinjiang	XJYLD181	Dog	2018	Cosmopolitan/ST	MK124744
China*	Xinjiang	XJYLD182	Dog	2018	Cosmopolitan/ST	MK124745
China*	Xinjiang	XLGLMC18	Cattle	2018	Cosmopolitan/ST	MK124740

Country	Location	Virus name	Species	Year	Phylogenetic clade/subclade	GenBank accession no.
China *	Zhejiang	ZJZSD1601	Dog	2016	Asian/SEA1	MG383893
China*	Zhejiang	ZJNBD18	Dog	2018	Asian/SEA1	MN784136
China	Anhui	HeF	Dog	1989	Asian/SEA1	HQ118104
China	Anhui	DRV-AH08	Dog	2008	Asian/SEA1	HQ450385
China	Anhui	11AF57	Dog	2011	Asian/SEA1	JQ798950
China	Beijing	BeijingHu1	Human	2007	Asian/SEA1	EU700031
China	Beijing	BJ16-13	Dog	2016	Asian/SEA1	KY214275
China	Beijing	BJ16-51	Dog	2016	Asian/SEA1	KY362747
China	Chongqing	CQ92	Dog	1992	Asian/SEA1	EU159388
China	Chongqing	CQH1202D	Dog	2012	Arctic-related/AL2	KM034905
China	Fujian	FJ004	Dog	2008	Cosmopolitan/Other	FJ561729
China	Fujian	FJ010	Dog	2008	Asian/SEA1	FJ866827
China	Guangdong	CGD0801D	Dog	2008	Asian/SEA2	JN974823
China	Guangxi	GX4	Dog	1994	Asian/SEA2	GU358653
China	Guangxi	N11	Dog	1997	Asian/SEA3	FJ594278
China	Guangxi	GXN119	Dog	2000	Asian/SEA3	DQ866111
China	Guangxi	GXLA	Dog	2003	Asian/SEA2	DQ866116
China	Guangxi	GX304	Dog	2004	Asian/SEA1	DQ866117
China	Guangxi	CGZ0501D	Dog	2005	Asian/SEA1	JN974830
China	Guangxi	GXSL	Cattle	2005	Asian/SEA2	DQ866120
China	Guangxi	CGX0601D	Dog	2006	Asian/SEA1	JN974825
China	Guangxi	CGX0602D	Dog	2008	Asian/SEA1	JN974826
China	Guangxi	CGX0801D	Dog	2008	Asian/SEA2	JN974829
China	Guizhou	Guizhou_A148	Dog	2004	Asian/SEA1	DQ666291
China	Guizhou	gk5	Dog	2006	Asian/SEA1	HQ118102
China	Heilongjiang	TJ11-RD	Raccoon dog	2011	Arctic-related/AL2	KM016898
China	Henan	Henan_Sq10	Dog	2004	Asian/SEA1	DQ666300
China	Henan	Henan_Sq35	Dog	2004	Asian/SEA1	DQ666304
China	Henan	Henan_Sq48	Dog	2004	Cosmopolitan/other	DQ666305
China	Hunan	Hunan_Wg22	Dog	2004	Cosmopolitan/other	DQ666310
China	Hunan	Hunan_Xx35	Dog	2004	Asian/SEA1	DQ666319
China	Hunan	HuNDN16	Dog	2005	Asian/SEA1	DQ515993
China	Hunan	CHN0635H	Human	2006	Asian/SEA2	JN974843
China	Hunan	CHN0504D	Dog	2008	Asian/SEA1	JN974837
China	Inner Mongolia	NeiMeng925	Raccoon dog	2007	Arctic-related/AL2	FJ415313
China	Inner Mongolia	NeiMeng927A	Raccoon dog	2007	Arctic-related/AL2	EU284093
China	Inner Mongolia	CNM1104D	Dog	2011	Asian/SEA1	KC465378

Country	Location	Virus name	Species	Year	Phylogenetic clade/subclade	GenBank accession no.
China	Inner Mongolia	DG11248	Dog	2013	Arctic-related	KT895593
China	Inner Mongolia	IMDRV-13	Deer	2013	Asian/SEA1	KJ564280
China	Inner Mongolia	WQ14	Cattle	2014	Cosmopolitan/ST	KM016901
China	Inner Mongolia	WQ15	Cattle	2015	Asian/SEA1	KU928249
China	Jiangsu	Jiangsu_Wx1	Dog	2004	Asian/SEA1	DQ666321
China	Jiangsu	CJS0847D	Dog	2008	Asian/SEA1	JN974852
China	Jiangxi	JX08-45	Ferret badger	2008	Asian/SEA2	GU647092
China	Jiangxi	CJX0901D	Dog	2009	Asian/SEA1	JN974853
China	Jiangxi	JX09-17 fb	Ferret badger	2009	Asian/SEA1	GU233765
China	Jiangxi	JX10-37	Ferret badger	2010	Asian/SEA1	KP319222
China	Jiangxi	JX11-05	Ferret badger	2011	Asian/SEA2	KP319193
China	Jiangxi	JX11-30	Ferret badger	2011	Asian/SEA1	KP319225
China	Jiangxi	JX12-150	Ferret badger	2012	Asian/SEA1	KP319228
China	Jiangxi	JX12-244	Ferret badger	2012	Asian/SEA2	KP319196
China	Jiangxi	JX13-09	Ferret badger	2013	Asian/SEA2	KP319201
China	Jiangxi	JX13-345	Ferret badger	2013	Asian/SEA1	KP319235
China	Jiangxi	JX13-418	Ferret badger	2013	Asian/SEA1	KP319220
China	Jiangxi	JX14-717	Ferret badger	2014	Asian/SEA1	KX447687
China	Jiangxi	JX14-719	Ferret badger	2014	Asian/SEA1	KX447689
China	Jiangxi	JX15-475	Ferret badger	2015	Asian/SEA1	KY613018
China	Ningxia	J	Human	1986	Asian/SEA1	GU345747
China	Ningxia	CNX1101H	Human	2011	Asian/SEA1	KC465379
China	Ningxia	NX15	Camel	2015	Asian/SEA1	KU928250
China	Shaanxi	CSX0903D	Dog	2009	Asian/SEA1	JN974876
China	Shaanxi	Shaanxi-HZ-6	Dog	2009	Asian/SEA1	KC977995
China	Shandong	CSD0709D	Dog	2007	Asian/SEA2	HM486381
China	Shanghai	SBD	Dog	1992	Asian/SEA1	EU159393
China	Shanghai	SBH	Human	1992	Asian/SEA1	EU159392
China	Shanghai	SH06	Dog	2006	Asian/SEA1	GU345748
China	Sichuan	CSC0808D	Dog	2008	Asian/SEA1	JN974857
China	Sichuan	Sichuan-BZ-1	Dog	2009	Asian/SEA1	GU591792
China	Taiwan	Nt2702	Ferret badger	2010	Asian/SEA5	KP860155
China	Taiwan	TW-1680	Ferret badger	2013	Asian/SEA5	KF501181
China	Taiwan	2014-3145	Ferret badger	2014	Asian/SEA5	KP892527
China	Tibet	CXZ1201D	Dog	2012	Arctic-related/AL2	KC465372
China	Tibet	CXZ1501H	Human	2015	Arctic-related/AL2	KY175230
China	Tibet	CXZ1704H	Human	2017	Indian Subcontinent	MH671332

Country	Location	Virus name	Species	Year	Phylogenetic clade/subclade	GenBank accession no.
China	Unknown	FY12	Dog	2005	Asian/SEA1	EU159372
China	Yunnan	CYN0818D	Dog	2008	Asian/SEA3	JF819603
China	Yunnan	CYN1009D	Dog	2010	Asian/SEA3	JX276405
China	Yunnan	CYN1134H	Human	2011	Asian/SEA2	JQ040592
China	Yunnan	CYN1247D	Dog	2012	Asian/SEA1	JX276411
China	Yunnan	CYN14114D	Dog	2014	Asian/SEA1	KP202447
China	Yunnan	Yunnan_Tc06	Dog	2006	Asian/SEA3	EU275243
China	Yunnan	Yunnan_Qj07	Dog	2007	Asian/SEA1	EU275245
China	Zhejiang	F02	Ferret badger	2008	Asian/SEA2	FJ712195
China	Zhejiang	F04	Ferret badger	2008	Asian/SEA2	FJ712196
China	Zhejiang	Zhejiang_Wz1_H	Human	2008	Asian/SEA1	EU700032
China	Zhejiang	ZJF4	Ferret badger	2008	Asian/SEA2	HQ118117
China	Zhejiang	ZJ-LA	Ferret badger	2008	Asian/SEA2	FJ598135
China	Zhejiang	D23	Dog	2010	Asian/SEA1	JX123685
China	Zhejiang	ZJ12-03	Ferret badger	2012	Asian/SEA1	KP319236
China	Zhejiang	ZJ13-66	Ferret badger	2013	Asian/SEA1	KP319237
India		ITN-R148	Dog	2005	Indian Subcontinent	KX434506
India		IAP-R192	Dog	2009	Arctic-related/AL1	KX434512
India		IAP-R194	Human	2011	Arctic-related/AL1	KX434514
India		IUP-R197	Equine	2012	Arctic-related/AL1	KX434517
India		IGU-201	Nilgai	2013	Arctic-related/AL1	KM099392
India		IGU-R202	Mongoose	2014	Arctic-related/AL1	KM099393
Indonesia		FL97-01	Dog	1997	Asian/SEA1	AB154218
Indonesia		SN00-14	Dog	2000	Asian/SEA1	AB154235
Indonesia		FL01-27	Dog	2001	Asian/SEA1	AB154222
Indonesia		SC01-65	Deer	2001	Asian/SEA1	AB154214
Indonesia		SC01-68	Cat	2001	Asian/SEA1	AB154208
Indonesia		SC01-70	Tiger	2001	Asian/SEA1	AB154242
Indonesia		SC01-75	Cat	2001	Asian/SEA1	AB154210
Indonesia		FL02-10	Dog	2002	Asian/SEA1	AB154217
Indonesia		SC02-87	Monkey	2002	Asian/SEA1	AB154224
Indonesia		SW02-22	Dog	2002	Asian/SEA1	AB154240
Indonesia		03003INDO	Dog	2003	Asian/SEA1	KX148266
Iran		87002IRA	Wolf	1984	Cosmopolitan/Other	KX148186
Iraq		RV2516	Cattle	2010	Cosmopolitan/Other	KF155000
Israel		93032ISR	Jackal	1993	Cosmopolitan/Other	KX148191
Japan		Komatsugawa	Dog	1940s	Arctic-related/AL2	AY352494

Country	Location	Virus name	Species	Year	Phylogenetic clade/subclade	GenBank accession no.
Kazakhstan		RV259	Red fox	1988	Cosmopolitan/ST	AY352491
Kazakhstan		4867	Cattle	2014	Cosmopolitan/ST	KT965738
Kazakhstan		5300	Jackal	2014	Cosmopolitan/other	KT965733
Kazakhstan		5303	Cat	2014	Cosmopolitan/ST	KT965734
Kazakhstan		5304	Cattle	2014	Cosmopolitan/ST	KT965735
Kazakhstan		5317	Dog	2014	Cosmopolitan/ST	KT965736
Kazakhstan		5328	Cattle	2014	Cosmopolitan/ST	KT965737
Laos		02001LAO	Dog	2002	Asian/SEA3	EU086194
Laos		Lao2	Dog	2011	Asian/SEA3	AB981663
Laos		Lao7	Dog	2011	Asian/SEA3	AB981668
Laos		Lao23	Dog	2012	Asian/SEA3	AB981677
Madagascar		04033MAD	Dog	2004	Cosmopolitan/ other	KX148209
Mexico		91010MEX	Mouse	1991	Cosmopolitan/ other	KX148112
Mongolia		MGL/34	Cat	2005	Cosmopolitan/ST	AB571016
Mongolia		MGL/35	Sheep	2005	Cosmopolitan/ST	AB571017
Mongolia		MGL/36	Dog	2005	Cosmopolitan/ST	AB571018
Mongolia		MGL/26	Cattle	2006	Cosmopolitan/ST	AB571008
Mongolia		MGL/28	Cattle	2006	Cosmopolitan/ST	AB571010
Mongolia		MGL-10	Cattle	2006	Cosmopolitan/ST	AB570997
Mongolia		MGL/22	Dog	2007	Arctic-related/AL2	AB571004
Mongolia		MGL/30	Cattle	2007	Cosmopolitan/ST	AB571012
Mongolia		MGL/25	Dog	2008	Cosmopolitan/ST	AB571007
Morocco		RV2627	Cattle	2009	Cosmopolitan/ other	KF155001
Myanmar		9909BIR	Dog	1999	Asian/SEA3	EU086164
Myanmar		9913BIR	Dog	1999	Asian/SEA3	EU086165
Myanmar		9915BIR	Dog	1999	Asian/SEA3	EU086166
Nepal		4403-13_09030NEP	Human	2003	Arctic-related/AL1	JX944565
Nepal		4403-17	Dog	2009	Indian Subcontinent	JX944597
Nepal		11016NEP	Dog	2011	Arctic-related/AL1	JX987742
Nepal		NPBUF12-1	Cattle	2012	Arctic-related/ AL1	KM979366
Pakistan		Pk 23	Mouse	2010	Arctic-related/AL1	HE802675
Philippines		94270PHI	Dog	1994	Asian/SEA4	EU086200
Philippines		94281PHI	Dog	1994	Asian/SEA4	KX148261
Philippines		03006PHI	Human	2000	Asian/SEA4	EU086203
Philippines		03007PHI	Human	2001	Asian/SEA4	EU086204
Russia		304c	Steppe fox	1977	Arctic-related/AL2	AY352459
Russia		857r	Raccoon dog	1980	Arctic-related/AL2	AY352458

Country	Location	Virus name	Species	Year	Phylogenetic clade/subclade	GenBank accession no.
Russia		686Cattle	Cattle	1989	Cosmopolitan/ST	AY352482
Russia		RV1596	Red fox	1990	Cosmopolitan/other	AY352474
Russia		RV245	Human	1990	Cosmopolitan/other	AY352475
Russia		RV1589	Cat	1991	Cosmopolitan/ST	AY352456
Russia		3561d	Dog	1996	Cosmopolitan/ST	AY352481
Russia		wolf-kras	Wolf	2002	Cosmopolitan/ST	JX423819
Russia		Rus_YamaloNenets_7405rdr	Deer	2006	Arctic-related/ AL1	KY002910
Russia		1350KRA	Dog	2008	Cosmopolitan/ST	JQ944705
Russia		1352KRA	Dog	2008	Cosmopolitan/other	JQ944706
Russia		1564NNO	Red fox	2008	Cosmopolitan/other	JQ944708
Russia		7445f	Fox	2008	Cosmopolitan/ST	KJ958248
Russia		7461f	Red fox	2008	Cosmopolitan/ST	KJ958249
Russia		7466Cattle	Cattle	2008	Cosmopolitan/ST	KJ958257
Russia		7499fA	Red fox	2008	Cosmopolitan/ST	KJ958231
Russia		7504f	Red fox	2008	Cosmopolitan/ST	KJ958237
Russia		7514f	Red fox	2008	Cosmopolitan/ST	KC794011
Russia		7548f	Red fox	2008	Cosmopolitan/ST	KC794014
Russia		Rus_YamaloNenets_7557af	Fox	2008	Arctic-related/ AL1	KY002909
Russia		7604c	Cat	2009	Cosmopolitan/ST	KJ958251
Russia		7891Cattle	Cattle	2011	Cosmopolitan/ST	KJ958225
Russia		7894w	Wolf	2011	Cosmopolitan/ST	KJ958228
Russia		8000f	Red fox	2011	Cosmopolitan/ST	KJ958254
Russia		8052f	Red fox	2011	Cosmopolitan/ST	KC595280
Russia		8057f	Red fox	2011	Cosmopolitan/ST	KC595283
Russia		8060c	Cat	2011	Cosmopolitan/ST	KJ958262
Russia		Rus_Altai_8041		2011	Cosmopolitan/ST	KY172632
Russia		Rus_Sakha/Yakutia_7985rdr	Deer	2011	Arctic-related/ AL1	KY002904
Russia		8061Cattle	Cattle	2012	Cosmopolitan/ST	KC538853
Russia		fox-2012	Red fox	2012	Cosmopolitan/ST	JX423818
Russia		wolf-bur	Wolf	2012	Cosmopolitan/ST	JX423817
Russia		PO-01_2014_Primorye	Bear	2014	Cosmopolitan/ST	KP997032
Russia		Kha-f1-15	Fox	2015	Cosmopolitan/ST	KY242672
Russia		Zbk_ctl2-15	Cattle	2015	Cosmopolitan/ST	KY242676

Country	Location	Virus name	Species	Year	Phylogenetic clade/subclade	GenBank accession no.
South Korea		BV9901PJ	Raccoon dog	1999	Arctic-related/AL2	KC171645
South Korea		SKRDG0203CW	Dog	2002	Arctic-related/AL2	DQ076124
South Korea		BD0406CC	Raccoon dog	2004	Arctic-related/AL2	KC171644
South Korea		KRH3-04	Dog	2004	Arctic-related/AL2	AY730596
South Korea		KRVB0910	Cattle	2009	Arctic-related/AL2	KJ476821
South Korea		KRVC1305	Dog	2013	Arctic-related/AL2	KF709096
Sri Lanka		H-1218-12	Human	2012	Indian Subcontinent	LC110310
Tajikistan		Tajik_FTA	Dog	2012	Cosmopolitan/ other	KY765901
Tanzania		RV2772	Dog	2010	Cosmopolitan/ other	KF155002
Thailand		8738THA	Human	1983	Asian/SEA3	EU086208
Thailand		8743THA	Human	1983	Asian/SEA3	EU086207
Thailand		THA1015	Dog	1985	Asian/SEA3	AB178893
Thailand		THA1017	Dog	1985	Asian/SEA3	AB178894
Turkey		93100TUR	Dog	1993	Cosmopolitan/other	KX148164
USA		CASK2	Striped skunk	1974	Cosmopolitan/other	JQ685970
USA		CA982	Striped skunk	1994	Cosmopolitan/other	JQ685894
USA		A10-0511	Gray fox	2009	Cosmopolitan/other	JQ685943
Vietnam		01017VNM	Dog	2001	Asian/SEA3	EU086210
		Flury_LEP	Vaccine	1985	Cosmopolitan/other	GU992324
		CVS-11	Vaccine	ND	Cosmopolitan/other	GQ918139
		SAD_Bern_Sanafox	Vaccine	ND	Cosmopolitan/other	EF206720
		PV-2061	Vaccine	ND	Cosmopolitan/other	JX276550
China (CN)		CTN-1	Vaccine	1956	Asian/SEA2	FJ959397

*Discussed in this study. ND, no date.

Appendix Table 4. Predicted transmission events of rabies virus subclades by BEAST

From	To	Bayes factor†	Posterior probability	Clade	Subclade
China (IM)	China (XJ)	229.87	0.98	Cosmopolitan	Steppe-type
China (XJ)	Kazakhstan	167.9	0.98	Cosmopolitan	Steppe-type
China (IM)	Russia	153.93	0.97	Cosmopolitan	Steppe-type
China (YN)	Thailand	84.99	0.94	Asian	SEA3
China	Indonesia	76.91	0.73	Asian	SEA1
Mongolia	China (HLJ)	15.59	0.75	Arctic-related	AL2
Russia	China (IM)	13.63	0.76	Cosmopolitan	Steppe-type
Russia	South Korea	6.85	0.56	Arctic-related	AL2
Mongolia	China (XZ)	5.93	0.53	Arctic-related	AL2
China (XJ)	China (IM)	5.81	0.58	Cosmopolitan	Steppe-type
Cambodia	China (YN)	5.35	0.5	Asian	SEA3
South Korea	Russia	5.2	0.5	Arctic-related	AL2
China (YN)	China (GX)	5.18	0.5	Asian	SEA3
Mongolia	China (IM)	4.97	0.49	Arctic-related	AL2
Cambodia	Myanmar	4.65	0.47	Asian	SEA3
Cambodia	China (GX)	4.61	0.47	Asian	SEA3
Kazakhstan	China (IM)	4.59	0.52	Cosmopolitan	Steppe-type
China (YN)	Myanmar	4.56	0.46	Asian	SEA3
Russia	China (XJ)	4.39	0.51	Cosmopolitan	Steppe-type
China (YN)	Cambodia	4.31	0.45	Asian	SEA3
Russia	Mongolia	4.11	0.49	Cosmopolitan	Steppe-type
China (XZ)	Mongolia	3.62	0.41	Arctic-related	AL2
China (IM)	Mongolia	3.41	0.39	Arctic-related	AL2
Laos	Vietnam	3.33	0.39	Asian	SEA3
Kazakhstan	Russia	3.18	0.43	Cosmopolitan	Steppe-type
Vietnam	Laos	3.17	0.38	Asian	SEA3
China (IM)	Mongolia	3.17	0.43	Cosmopolitan	Steppe-type

*GX, Guangxi ; HLJ, Heilongjiang; IM, Inner Mongolia; XJ, Xinjiang; XZ, Tibet; YN, Yunnan.

†Bayes factor was used to determine the best supported transmission event between two geographic locations, which is calculated by SpreaD3 software with a value >3 as the cutoff value.
